# Alpha-pinene modulates feeding behavior and hypothalamic orexin-A expression in a rat model of painful temporomandibular disorder

**DOI:** 10.22514/jofph.2026.019

**Published:** 2026-03-12

**Authors:** Hanieh Eghbali, Mehdi Abbasnejad, Maryam Raoof, Mahnaz Zamyad, Saeed Esmaeili-Mahani, Razieh Kooshki, Mojdeh Mansoori, Frank Lobbezoo

**Affiliations:** ^1^Department of Biology, Faculty of Sciences, Shahid Bahonar University of Kerman, 76169-14111 Kerman, Iran; ^2^Laboratory of Molecular Neuroscience, Kerman Neuroscience Research Center (KNRC), Kerman University of Medical Sciences, 76198-13159 Kerman, Iran; ^3^Department of Orofacial Pain and Dysfunction, Academic Centre for Dentistry Amsterdam (ACTA) University of Amsterdam and Vrije Universiteit Amsterdam, 1081 LA Amsterdam, The Netherlands; ^4^Department of Biology, Faculty of Sciences, Lorestan University, 68151-44316 Khorramabad, Iran; ^5^Section for Orofacial Pain and Jaw Function, Department of Dentistry and Oral Health, Aarhus University, 8000 Aarhus, Denmark; ^6^Department of Orofacial Pain and Jaw Function, Faculty of Odontology, Malmö University, 205 06 Malmö, Sweden

**Keywords:** Temporomandibular joint disorders, Alpha-pinene, Feeding behavior, Orexins, Rats

## Abstract

**Background**: Temporomandibular disorders (TMDs) are common conditions involving the temporomandibular joint (TMJ) and masticatory muscles, often presenting with pain and impaired orofacial function. Painful TMD can disrupt jaw motor activities, including chewing and feeding behavior, reflecting alteration in muscle performance and central neuroregulation. The hypothalamic neuropeptide orexin A integrates pain, arousal, and energy balance and may be involved in these disturbances. This study examined whether intracerebroventricular (ICV) administration of alpha-pinene, an anti-inflammatory monoterpene, could modulate pain-related impairments in feeding behavior and orexin A expression in a rat model of inflammatory TMD. **Methods**: TMJ inflammation was induced in male Wistar rats via Complete Freund’s Adjuvant (CFA) injection. Rats received ICV alpha-pinene (0.1, 0.2, or 0.4 μg/rat). Feeding behavior parameters—including meal frequency, duration, and total intake—were recorded with an automated monitoring system as functional readouts of masticatory muscle activity during food processing. Anxiety-like behavior was evaluated using the elevated plus maze, and hypothalamic orexin A expression was assessed by immunohistochemistry. **Results**: CFA-treated rats showed reduced pain thresholds, anxiety-like behavior, and impaired feeding behavior, including fewer meals, shorter feeding duration, and reduced intake. Alpha-pinene, particularly at 0.4 μg/rat, significantly improved these behavioral outcomes and restored hypothalamic orexin A expression compared with untreated CFA rats. **Conclusions**: Alpha-pinene mitigated pain-related disruptions in feeding behavior and restored hypothalamic orexin A expression in a rat model of TMJ inflammation. These findings highlight the interplay between orofacial pain, altered oral motor function, and central neuroregulation. The observed behavioral improvements suggest that alpha-pinene may offer therapeutic benefits for managing functional impairments associated with both muscular and joint-related TMD pain, supporting its potential as a candidate for integrative TMD management.

## 1. Introduction

Temporomandibular disorders (TMDs) are a heterogeneous group of conditions affecting the temporomandibular joint (TMJ), masticatory muscles, and associated orofacial structures. Painful TMD is the second most common cause of chronic orofacial pain [1] and often involves impairments in muscle coordination, jaw mobility, and oral motor activities. Among others, these disorders manifest as difficulty in chewing, prolonged meal duration, and reduced food intake, especially when movements such as biting or sustained mastication exacerbate discomfort [2]. Beyond nociception, TMD-related pain disrupts coordinated orofacial muscle activity, interfering with essential behaviors such as feeding and contributing to altered dietary patterns [3]. For the induction of pain in the TMJ as an experimental model, chemical methods such as the injection of Complete Freund’s Adjuvant (CFA) into the joint are well-established approaches.

Models of TMD based on CFA-induced TMJ inflammation reliably replicate key features of the clinical condition, including local inflammation, nociceptive hypersensitivity, and behavioral signs of pain [4, 5, 6, 7]. These models also provide a valuable window into how orofacial muscle activity, especially during feeding, is impaired under inflammatory pain conditions. Reduced jaw excursions, altered chewing rhythms, and fatigue-related changes in muscle endurance are among the documented consequences of TMD in both clinical and experimental settings [2, 8, 9]. Moreover, orofacial pain may interfere with higher-order cognitive functions such as decision-making and working memory, which may secondarily disrupt food-seeking behaviors [10].

The hypothalamic orexinergic system plays a pivotal role at the interface between pain regulation, energy balance, and oromotor activity. Orexin A and B, neuropeptides produced in the lateral hypothalamus, act on orexin receptors (OX1 and OX2) with dense projections to pain-modulatory areas in the brainstem, such as the periaqueductal gray, parabrachial nucleus, and raphe nuclei [11, 12, 13, 14, 15]. Activation of these circuits can reduce trigeminal nociceptive signaling and modulate pain-induced alterations in feeding and locomotor behaviors [13, 16, 17, 18]. Disruption of this system by inflammatory pain may underlie the motivational and behavioral changes observed in chronic TMD [19, 20, 21, 22, 23].

Alpha-pinene, a bicyclic monoterpene found in the essential oils of numerous plants, including *Rosmarinus officinalis*, has emerged as a promising candidate in pain research due to its multi-target properties. It exhibits anti-inflammatory, neuroprotective, and antioxidant activities, with evidence for downregulation of nuclear factor kappa-light-chain-enhancer of activated B cells (NF-κB) and mitogen-activated protein kinase (MAPK) pathways [24, 25], attenuation of cytokine expression—interleukin-1β (IL-1β) and tumor necrosis factor-α (TNF-α), and suppression of cyclooxygenase-1 (COX-1) in the spinal cord [26]. Notably, alpha-pinene has been shown to interact with gamma-aminobutyric acid (GABA)-ergic and μ-opioid receptors [27, 28, 29], reduce neuroinflammation, and promote anxiolytic-like behaviors [30, 31]. These actions may alleviate both nociceptive transmission and muscle tension, key components in pain-related orofacial motor dysfunction.

Emerging research also suggests peripheral mechanisms through which alpha-pinene may enhance muscle function. For example, it promotes glucose transporter-4 (GLUT4) gene expression and membrane translocation in skeletal muscle cells, improving glucose uptake and muscle energetics [32]. Furthermore, alpha-pinene and its metabolites induce nitric oxide (NO)-mediated vasorelaxation in mesenteric arteries via endothelial nitric oxide synthase (eNOS) activation, enhancing tissue perfusion [33]. These properties may be particularly relevant in conditions like TMD, where jaw-muscle fatigue and impaired blood flow can exacerbate functional decline [13, 34].

Despite the extensive evidence supporting alpha-pinene’s biological activity, its effects on orofacial muscle performance and associated behaviors such as feeding, have not been well explored in inflammatory pain models. The present study aimed to address this gap by investigating whether ICV administration of alpha-pinene could modulate CFA-induced alterations in feeding behavior and hypothalamic orexin A expression in rats with TMJ inflammation. We hypothesized that alpha-pinene would attenuate pain-related impairments in feeding behavior and enhance hypothalamic orexin A expression in this model.

## 2. Materials and methods

### 2.1 Animals

This experimental study included 42 adult male Wistar rats (Pasteur Institute, Tehran, Iran) divided six groups (n = 7 per group), weighing 200–250 g. The sample size was determined based on previous similar studies [18, 19]. Animals were housed under controlled conditions (12-h light/dark cycle, 23 ± 2 °C) with free access to standard chow and water. To minimize stress and variability in feeding behaviors, rats were acclimatized to the laboratory environment and apparatus for 15 minutes before testing. Each animal was used only once for all test protocols. All procedures were approved by the Institutional Ethics Committee of Shahid Bahonar University of Kerman (IR.UK.VETMED.REC.1401.019).

### 2.2 Experimental groups

Rats were randomly assigned to the following groups:

• Control: no surgical intervention or drug administration.

• CFA: unilateral injection of Complete Freund’s Adjuvant (CFA) into the TMJ after ICV cannulation.

• Vehicle: ICV and TMJ injections with normal saline.

• Alpha-pinene: CFA injection into the TMJ, followed by ICV administration of alpha-pinene (0.1, 0.2, or 0.4 μg/rat).

### 2.3 TMJ inflammation procedure

Rats were anesthetized, and bilateral ICV cannulation was performed. After recovery, unilateral CFA (20 μL) (Flcm7415, Sigma-Aldrich, St. Louis, MO, USA) was injected into the left TMJ to induce pain. Inflammation and pain were confirmed by swelling in the TMJ region, periorbital edema, mandibular movement, facial rubbing, and reduced feeding activity. Pain symptoms typically persisted for several days, reflecting sustained orofacial dysfunction. Alpha-pinene (177524-5, Sigma-Aldrich, St. Louis, MO, USA) was administered via ICV injection (0.1, 0.2, or 0.4 μg/rat) after a 7-day recovery period. Following behavioral assessments, rats were perfused, and brains were processed for immunohistochemical analyses. Fig. 1 illustrates the study procedure and timeline.

**Fig. 1.** Timeline of the study. ICV: intracerebroventricular; CFA: Complete Freund’s Adjuvant; EPM: Elevated plus maze; IHC: Immunohistochemistry.

### 2.4 Stereotaxic surgery procedure 

Rats were anesthetized with ketamine (60 mg/kg; batch number: 1705225-02) and xylazine (5 mg/kg; 0906167-10, Alfasan, Woerden, The Netherlands) and fixed in a stereotaxic apparatus (Stoelting, Co-620, Wood Dale, IL, USA). Stainless-steel guide cannulas (23-gauge) were bilaterally implanted into the lateral ventricles using stereotaxic coordinates from the Paxinos and Franklin atlas (anteroposterior (AP) = 1.6 mm, mediolateral (ML) = ±0.8 mm, dorsoventral (DV) = 3.4 mm) [10]. Cannulas were anchored with screws and dental acrylic. Animals recovered for seven days in individual cages before experiments commenced [35].

### 2.5 Feeding behavior apparatus

Feeding behavior was recorded using an automated feeding and drinking monitoring system [36]. The apparatus registered the number of food container visits using infrared sensors, while weight sensors beneath the containers measured the amount of food consumed. These parameters served as indirect indicators of pain-related changes in oral motor function, such as reduced or cautious feeding behavior, commonly observed in orofacial pain models. The time spent near food sources and locomotor activity were also tracked using symmetrical weight sensors under the apparatus. Animals were tested individually in the feeding behavior apparatus.

### 2.6 Nutritional variables assessment

After one week of recovery from surgery, rats underwent a 12-h fasting period prior to testing. Standard pellets (15 g) were provided, and each animal was allowed to freely explore the apparatus for 12 h. Data collected included total food and water intake, frequency of food visits, time spent feeding, and distance traveled. These variables reflected potential alterations in feeding and food-acquisition behavior due to TMJ inflammation and pain.

### 2.7 Elevated plus maze (EPM)

Anxiety-like behavior was assessed using a standard wooden EPM with two open and two closed arms (50 cm each; central platform 10 × 10 cm; height 60 cm). Each rat was placed in the center facing an open arm and observed for 5 minutes. Time spent in and entries into open versus closed arms were recorded, with increased open-arm exploration interpreted as reduced anxiety. The maze was disinfected with 70% alcohol between trials [37].

### 2.8 Hotplate test

Thermal nociception was assessed 24 h after CFA injection using the hot plate test. Rats were placed on a hotplate maintained at 52 ± 0.5 °C, with a 30-s cutoff to prevent tissue damage. Behavioral responses, such as hind-paw licking, rearing, or jumping, were recorded as nociceptive endpoints. The maximum possible effect (MPE%) was calculated as (Eqn. 1):

(1) $$\mathrm{MPE}\%\;=\;\left[\frac{\left(\mathrm{post}\text{-}\mathrm{drug}\;\mathrm{latency}\;\text{−}\;\mathrm{baseline}\;\mathrm{latency}\right)}{\left(60\;\text{−}\;\mathrm{baseline}\;\mathrm{latency}\right)}\right]\;\times 100$$

Where post-drug latency = reaction time (s) after administration of alpha-pinene or vehicle; baseline latency = reaction time (s) before any treatment; and 60 = cutoff time (s) [38].

### 2.9 Immunohistochemistry (IHC)

Rats were anesthetized with 10% ketamine and 2% xylazine. A thoracic incision exposed the heart, and a catheter was inserted into the left ventricle, followed by systemic perfusion with 10% formalin. After fixation, brains were removed and post-fixed in 10% formalin. Paraffin blocks were prepared, and 3-μm sections were cut with a microtome. Sections were oven-dried at 60 °C for 1 h, cleared twice in xylene (5 min each), and dehydrated through graded ethanol (100%, 96%, 70%, 50%; 1 min each). After rinsing with Tris-buffered saline with Tween-20 (TBST), antigen retrieval was performed. Sections were incubated overnight with primary antibodies, followed by peroxidase-conjugated secondary antibodies. Immunoreactivity was visualized with 3,3′-diaminobenzidine (DAB; 5–15 min). Hematoxylin was used for nuclear counterstaining. After washing, dehydration, and clearing, slides were mounted and cover-slipped for microscopic analysis [39].

### 2.10 Histopathology

Samples were fixed in 10% formalin for one week and processed in a tissue preparation device. Paraffin blocks were prepared, and 5-μm sections were mounted on slides and incubated at 70 °C for 30 minutes. Sections were cleared in xylene (30 min) and fully deparaffinized. After hydration through descending alcohols (100%, 96%, 80%, 70%), hematoxylin and eosin staining was performed. Sections were dehydrated with ascending alcohols (70%, 80%, 96%, 100%) and cleared in xylene (30 min). Slides were mounted with Entellan and examined under a light microscope at 40× and 100× magnifications [40].

### 2.11 Statistical analyses

Data are presented as mean ± Standard error of the mean (SEM). The normality of the data was assessed using the Kolmogorov-Smirnov test. Statistical comparisons among groups were made using one-way analysis of variance (ANOVA), followed by Tukey’s *post hoc* test to explore differences between groups. A *p*-value < 0.05 was considered statistically significant. Analyses were performed using SPSS version 21 (IBM Corp., Armonk, NY, USA).

## 3. Results

### 3.1 Pain behavior assessment

The rat grimace scale (RGS) was used as a macroscopic indicator of TMJ inflammation. Measurements of the distance between the eyes and between the ears showed significant changes in CFA-treated rats compared with controls, indicating facial swelling and pain-associated orofacial dysfunction (Fig. 2). These alterations were consistent with visible signs of discomfort during mandibular movements.

**Fig. 2.** Differences in interocular and interaural distances between healthy rats and rats with CFA-induced TMJ inflammation. Data are presented as mean ± standard error of the mean. CFA: Complete Freund’s Adjuvant. ***p  < 0.001 versus control.

### 3.2 Hot plate test

As shown in Fig. 3, the CFA and CFA + saline groups exhibited significantly shorter response latencies to the thermal stimulus compared with the control group (*p * < 0.01), confirming the presence of hyperalgesia. Treatment with alpha-pinene at 0.2 and 0.4 μg/rat significantly prolonged response latency relative to the CFA group, indicating attenuation of pain sensitivity. One-way ANOVA confirmed this observation (*F*(5, 36) = 18.25, *p * < 0.0001).

**Fig. 3.** Response latency to the thermal stimulus in the hot plate test across experimental groups. Data are presented as mean ± standard error of the mean. CFA: Complete Freund’s Adjuvant. **p  < 0.01 versus control; ##*p * < 0.01, #*p * < 0.05 versus CFA.

### 3.3 Assessment of anxiety-like behaviors

Fig. 4a demonstrates that time spent in the open arms of the elevated plus maze was significantly reduced in the CFA and CFA + saline groups compared with the control group (*p * < 0.001), reflecting pain-induced anxiety. Alpha-pinene treatment at 0.4 μg/rat significantly reversed this effect. One-way ANOVA confirmed this observation (*F*(5, 36) = 19.36, *p * < 0.0001). Similarly, the number of entries into open arms (Fig. 4b) was significantly decreased in the CFA and CFA + saline groups compared with the control group (*p * < 0.001) but increased significantly after alpha-pinene treatment at 0.2 and 0.4 μg/rat (*p * < 0.05, *p * < 0.001, respectively). One-way ANOVA confirmed this observation (*F*(5, 36) = 10.76, *p * < 0.0001).

**Fig. 4.** Evaluation of (a) time spent in the open arms and (b) the number of entries into open arms in the elevated plus maze test among experimental groups. Data are presented as mean ± standard error of the mean. CFA: Complete Freund’s Adjuvant. ***p  < 0.001 versus control; ###*p * < 0.001 versus CFA; ^^*p * < 0.01 versus CFA + Saline; *p  < 0.05 versus control; #*p * < 0.05 versus CFA.

### 3.4 Feeding behaviors

TMJ inflammation significantly altered feeding behavior patterns (Fig. 5a–c).

**Fig. 5.** Feeding behavior parameters in experimental groups. (a) Number of visits to the food container; (b) total food consumption; (c) duration of food intake. Data are presented as mean ± standard error of the mean. CFA: Complete Freund’s Adjuvant. ***p  < 0.001 versus control; ###*p * < 0.001, #*p * < 0.05 versus CFA; +++*p * < 0.001 versus CFA + Alpha-pinene (0.4 μg/rat); *p < 0.05 versus control; ^^^*p * < 0.001 versus CFA + Saline.

• Meal frequency: significantly decreased in the CFA and CFA + saline groups compared with controls (*p * < 0.001). Alpha-pinene treatment increased frequency at 0.2 and 0.4 μg/rat (*p * < 0.05, *p * < 0.001, respectively) (Fig. 5a). One-way ANOVA: (*F*(5, 36) = 54.56, *p * < 0.0001).

• Food consumption: total intake was significantly reduced in the CFA (*p * < 0.001) and CFA + saline (*p *< 0.01) groups compared with the control group. Treatment with alpha-pinene at 0.4 μg/rat increased consumption compared with the CFA group (*p * < 0.001) (Fig. 5b). One-way ANOVA: *F*(5, 36) = 21.52, *p * < 0.0001.

• Feeding duration: significantly reduced in the CFA and CFA + saline groups compared with controls (*p * < 0.001). Alpha-pinene at 0.2 and 0.4 μg/rat significantly increased feeding duration (*p* < 0.05, *p * < 0.001, respectively) (Fig. 5c). Additionally, significant differences were observed between the lower doses (0.1 and 0.2 μg/rat) and 0.4 μg/rat (*p * < 0.001). One-way ANOVA: *F*(5, 36) = 33.67, *p * < 0.0001.

### 3.5 Histopathology

Control rats showed normal TMJ morphology with normal intercellular space and intact joint capsule (Fig. 6a). CFA-treated rats exhibited signs of inflammation, including increased intercellular spacing and hemorrhage within the TMJ tissue (Fig. 6b), confirming localized inflammatory changes associated with altered orofacial function.

**Fig. 6.** Histopathology of the TMJ region. (a) Control rats: circle, normal intercellular space; star, intact joint capsule. (b) CFA-induced inflammation: circle, expanded intercellular space; arrow, hemorrhage in jaw tissue; asterisk, TMJ capsule.

### 3.6 Immunohistochemical evaluation of orexin A expression

Orexin A immunoreactivity in the hypothalamus was significantly reduced in CFA-treated animals compared to controls (Fig. 7), indicating a central neurochemical alteration associated with pain and impaired feeding behavior. Treatment with alpha-pinene at 0.4 μg/rat significantly increased orexin A expression relative to CFA alone, suggesting partial restoration of hypothalamic regulation of feeding behavior and pain-modulation pathways.

**Fig. 7.** Immunohistochemical expression of orexin A in the hypothalamus of control, CFA-treated, and alpha-pinene (0.4 μg/rat)-treated rats. CFA: Complete Freund’s Adjuvant.

A summary of the behavioral, histological, and neurochemical outcomes across experimental groups is presented in Table 1.

**Table 1.** Summary of behavioral, histological, and neurochemical outcomes across experimental groups.

Parameter	Group Comparison	Main Findings	Statistical Results
Pain behavior (Rat Grimace Scale)	CFA *vs. *Control	CFA induced facial swelling, with increased interaural and interocular distances, indicating orofacial pain and inflammation.	*p *< 0.001 *vs. *control
Thermal nociception (Hot Plate Test)	CFA, CFA + Saline *vs. *Control; α-Pinene (0.1, 0.2, 0.4 µg/rat) *vs.* CFA	CFA and CFA + saline groups exhibited decreased latency to thermal stimulus (hyperalgesia). α-Pinene, particularly at 0.4 µg/rat, significantly increased latency, indicating antinociceptive activity.	One-way ANOVA: *F*(5, 36) = 18.25, *p * < 0.0001
Anxiety-like behavior (Elevated Plus Maze)	CFA, CFA + Saline *vs.* Control; α-Pinene (0.1, 0.2, 0.4 µg/rat) *vs.* CFA	CFA reduced time spent and entries into open arms, reflecting increased anxiety-like behavior. α-Pinene (0.4 µg/rat) significantly reversed these effects.	*F*(5, 36) = 19.36, *p * < 0.0001 (time); *F*(5, 36) = 10.76, *p * < 0.0001 (entries)
Feeding behavior	CFA, CFA + Saline *vs. *Control; α-Pinene (0.1, 0.2, 0.4 µg/rat) *vs.* CFA	CFA markedly reduced meal frequency, food intake, and feeding duration. α-Pinene restored all feeding parameters in a dose-dependent manner.	*F*(5, 36) = 54.56, 21.52, 33.67; all *p * < 0.0001
Histopathology (TMJ)	CFA *vs.* Control	CFA caused increased intercellular spacing and hemorrhage within TMJ tissue. Control rats showed normal joint morphology.	Descriptive (qualitative)
Orexin A immunoreactivity (Hypothalamus)	CFA *vs.* Control; α-Pinene (0.4 µg/rat) *vs.* CFA	CFA markedly decreased hypothalamic orexin A expression. α-Pinene (0.4 µg/rat) restored orexin A immunoreactivity, suggesting neurochemical recovery.	Semi-quantitative (visual comparison)

*CFA: Complete Freund’s Adjuvant; TMJ: temporomandibular joint; ANOVA: analysis of variance.*

## 4. Discussion

This study investigated the effects of ICV alpha-pinene on feeding behavior and hypothalamic orexin A expression in a rat model of CFA-induced TMJ inflammation, a well-established experimental proxy for painful TMD. As expected, CFA treatment produced significant nociceptive hypersensitivity, as evidenced by reduced latency in the hot plate test and facial swelling, consistent with prior studies on orofacial inflammatory pain [4, 5, 7, 13].

In addition to increased pain sensitivity, CFA-treated rats demonstrated reduced exploratory behavior in the elevated plus maze and significant impairments in feeding behavior, including lower meal frequency, reduced intake, and shorter feeding durations. These changes likely reflect pain-related limitations in orofacial function, possibly due to restricted jaw movement or discomfort during mastication. Alpha-pinene administration, particularly at 0.4 μg/rat, ameliorated these deficits, improving both behavioral and functional outcomes. These behavioral improvements suggest an underlying neurobiological mechanism, which was further supported by histological analysis of the hypothalamus.

Histological analysis showed reduced orexin A expression in the hypothalamus of CFA-treated rats, a change significantly reversed by alpha-pinene. Given orexin A’s established roles in modulating nociception, arousal, and feeding behavior [12, 13, 15], these findings suggest that TMJ inflammation alters orexinergic signaling, and that alpha-pinene may restore orexin-dependent regulatory functions. This aligns with the hypothesis that orofacial pain disrupts both central motivational circuits and peripheral behaviors relevant to feeding behavior. The restoration of orexinergic signaling by alpha-pinene is likely attributable to its potent anti-inflammatory properties at the molecular level.

CFA-induced inflammation involves activation of immune responses and cytokine cascades, including NF-κB signaling [5, 7, 33], which contribute to both pain and functional deficits. Alpha-pinene has been shown to inhibit inflammatory signaling through MAPK and NF-κB pathways [24, 25], reduce cytokine production (IL-1β, TNF-α), and downregulate COX-1 in models of inflammatory pain [26]. Beyond these broad anti-inflammatory effects, alpha-pinene also exhibits specific neuroprotective and metabolic properties.

Previous studies have shown that alpha-pinene improves memory and antioxidant status [30, 31, 41], modulates pain through GABAergic and μ-opioid pathways [27, 28, 29], and enhances GLUT4 expression and membrane translocation in skeletal muscle [32], promoting glucose uptake and delaying muscle fatigue. These mechanisms may help explain the improved feeding behavior observed following alpha-pinene administration.

Emerging evidence suggests that cytokine signaling, particularly via IL-1 and IL-6, plays a key role in regulating glucose metabolism and endurance in masticatory muscles. Chiba *et al.* [34] demonstrated that disruption of IL-1 signaling impairs GLUT4 function and induces masseter muscle fatigue. Therefore, the improved feeding behavior in alpha-pinene-treated rats may indirectly reflect enhanced oral motor endurance and reduced cytokine-mediated fatigue. Complementing this potential for improved muscular endurance, alpha-pinene may also enhance functional recovery by improving blood flow to the orofacial region.

In addition to modulating inflammation and metabolism, alpha-pinene may enhance orofacial tissue perfusion. Jin *et al*. [33] showed that alpha-pinene induces vasorelaxation via NO-mediated pathways, potentially improving blood flow. Improved perfusion could contribute to functional recovery during pain. These proposed physiological mechanisms are reflected in the complex interplay between feeding behavior and pain perception observed in both clinical and experimental settings.

The link between mastication and pain modulation is further supported by studies showing transient pain suppression during feeding, possibly via activation of brainstem inhibitory pathways [2]. Clinically, patients with TMD or other orofacial pain conditions often modify their eating behavior, *e.g.*, prolonging meals or reducing bite force to manage their pain [9, 42]. This adaptation is mirrored in CFA-treated rats, which exhibited decreased feeding frequency and shorter meal durations, a pattern that was partially reversed by alpha-pinene. Critically, this behavioral recovery was accompanied by a corresponding neurochemical restoration in the hypothalamus.

Restoration of feeding behavior was accompanied by increased hypothalamic orexin A expression. Since orexins regulate both nociception and feeding behavior [16, 17, 18, 19, 21, 22, 23], this suggests that alpha-pinene may act not only through anti-inflammatory pathways but also by enhancing central orexinergic signaling disrupted by chronic pain.

Together, these findings demonstrate that alpha-pinene attenuates CFA-induced orofacial pain and supports the recovery of feeding behavior under inflammatory conditions. Alpha-pinene’s multimodal actions; anti-inflammatory, metabolic, vasodilatory, and neuromodulatory; make it a promising candidate for managing pain conditions that impair feeding behavior. This study highlights how pain alters orofacial function and identifies phytochemical interventions that may restore feeding behaviors. These insights underscore the value of interdisciplinary approaches to orofacial pain management, integrating behavioral, neuropharmacological, and physiological perspectives.

## 5. Strengths and limitations

A strength of this study is the integration of behavioral, histological, and neurochemical outcomes to investigate the effects of alpha-pinene in a validated animal model of TMD pain. A limitation is that masticatory muscle activity was not directly assessed with electromyography (EMG). Nevertheless, the focus on feeding behavior provided a clinically meaningful readout of orofacial motor function, capturing the integrated impact of pain on oral behavior rather than isolated muscle activity. Future studies may benefit from combining EMG recordings with behavioral assessments to provide a more comprehensive evaluation of orofacial muscle function under painful conditions.

## 6. Conclusions

Alpha-pinene attenuated pain behaviors and feeding behavior disturbances in a rat model of TMJ inflammation, likely through restoration of hypothalamic orexin A expression. These findings suggest that alpha-pinene modulates orofacial pain and function via central orexinergic pathways. With its combined anti-inflammatory, vasodilatory, metabolic, and GABAergic actions, alpha-pinene emerges as a multi-target phytochemical with potential relevance for both myogenic and arthrogenic forms of TMD pain. Its ability to promote muscle relaxation and restore feeding behaviors underscores its therapeutic promise. Future studies should clarify its specific effects on muscle- and joint-related TMD pain and explore optimized delivery strategies, such as nanoemulsions. Before translation to clinical practice, additional work is required to establish pharmacokinetics, dosing, safety, and efficacy in both preclinical and clinical settings.
